# The International Encyclopædia of Surgery

**Published:** 1884-03

**Authors:** 


**The International Encyclopædia of Surgery:** A Systematic Treatise of the Theory and Practice of Surgery by Authors of Various Nations. Edited by John Ashhurst, Jr., M. D., Professor of Clinical Surgery in the University of Pennsylvania. Illustrated with Chromo-Lithographs and Wood Cuts. In six volumes. Volume iv. William Wood & Co.: New York. 1884.

The fourth volume of this work concludes the articles on Injuries and Diseases of the various tissues, and with it commences the discussion of the Surgery of Regions. Of these the article on diseases of bones, to be written by Prof. Allier, of Lyons, is postponed to a later portion for the reason that it has been found unable to prepare it for this.

From the preface we also extract the following: " The elaborate article herewith presented on Injuries of the Back possesses the melancholy interest of being a posthumous contribution from the pen of the lamented Lidell, who died almost as the last proof-sheets of his work were being corrected." It had been his intention to add a brief supplement, referring to several cases of spinal injury recorded in the third surgical volume of the Medical and Surgical History of the War of the Rebellion, and the preface further adds, that the author " would doubtless also have taken some notice of the recently published views of Mr. H. W. Page, as to the so-called ' Rai way Injuries of the Spine,' views which differ in certain particulars from those which Dr. Lidell has advocated."

The list of articles contained in the fourth volume is as follows:

Injuries of Bones, by John H. Packard, M. D., Surgeon of the Episcopal Hospital and St. Joseph Hospital, Philadelphia; Diseases of the Joints, by Richard Barwell, F. R. C. S., Surgeon to Charing Cross Hospital, London; Excisions and Resections, by John Ashhurst, Jr., M. D., Professor of Clinical Surgery in the University of Pennsylvania; Excision of the Knee Joint, by George E. Fennick, M. D., C. M., Professor of Surgery in McGill University, Surgeon to the Montreal General Hospital; Tumors, by Henry Treatham Butler, F. R. C. S., Assistant Surgeon to St. Bartholomew's Hospital, London; Injuries of Back and Spinal Column, Cord, etc., by John Lidell, A. M., M. D., late Surgeon to Bellevue Hospital, New York, etc.; Malformations and Diseases of the Spine, by Frederick Treves, F. R. C. S., Assistant Surgeon to London Hospital.

The volume contains six chromo-lithographs and two hundred and twenty-six wood-cuts.

Our readers need not be told that the names of the contributors above rehearsed are a guarantee of the character of the work. In its scope and purpose it compares with the well-known Holmes System of Surgery, and it forms as complete and authoritative a work of reference as any with which we are familiar. The style of the volume, comprising nearly a thousand pages of elegantly printed matter, as well as the superior binding is an example of the best work of the publishers, William Wood & Co., who have for many years done so much for medical literature. As we have implied, the work is one for reference, comprising, as it will when completed, six volumes of nearly equal size. It would appear that for some time, at least until new discoveries and researches again change the science of surgery in some of its branches, that nothing more could be desired by the surgeon than he will have upon the completion of the present Encyclopædia of Surgery.

